# RETRACTION: Intrawound Vancomycin Powder in Orthopaedic Surgery as Surgical Site Wound Infection Prophylaxis: A Meta‐Analysis

**DOI:** 10.1111/iwj.70205

**Published:** 2025-02-02

## Abstract

**RETRACTION:** WangB. , LiS. , ZhangJ. , WuD. , HuangX. , LiuD. , and DuJ. , “Intrawound Vancomycin Powder in Orthopaedic Surgery as Surgical Site Wound Infection Prophylaxis: A Meta‐Analysis,” International Wound Journal 20, no. 9 (2023): 3673–3681, 10.1111/iwj.14258.37309291 PMC10588340

The above article, published online on 12 June 2023, in Wiley Online Library (http://onlinelibrary.wiley.com/), has been retracted by agreement between the journal Editor in Chief, Professor Keith Harding; and John Wiley & Sons Ltd. Following an investigation by the publisher, all parties have concluded that this article was accepted solely on the basis of a compromised peer review process. The editors have therefore decided to retract the article. The authors did not respond to our notice regarding the retraction.

**RETRACTION:** B. Wang , S. Li , J. Zhang , D. Wu , X. Huang , D. Liu , and J. Du , “Intrawound Vancomycin Powder in Orthopaedic Surgery as Surgical Site Wound Infection Prophylaxis: A Meta‐Analysis,” International Wound Journal 20, no. 9 (2023): 3673–3681, 10.1111/iwj.14258.37309291 PMC10588340

The above article, published online on 12 June 2023, in Wiley Online Library (http://onlinelibrary.wiley.com/), has been retracted by agreement between the journal Editor in Chief, Professor Keith Harding; and John Wiley & Sons Ltd. Following an investigation by the publisher, all parties have concluded that this article was accepted solely on the basis of a compromised peer review process. The editors have therefore decided to retract the article. The authors did not respond to our notice regarding the retraction.

